# Vinorelbine Alters lncRNA Expression in Association with EGFR Mutational Status and Potentiates Tumor Progression Depending on NSCLC Cell Lines’ Genetic Profile

**DOI:** 10.3390/biomedicines11123298

**Published:** 2023-12-13

**Authors:** Hasan Alsharoh, Paul Chiroi, Andreea Nutu, Lajos Raduly, Oana Zanoaga, Ioana Berindan-Neagoe

**Affiliations:** Research Center for Functional Genomics, Biomedicine and Translational Medicine, “Iuliu Hatieganu” University of Medicine and Pharmacy, 400337 Cluj-Napoca, Romania; hasanalsharoh@gmail.com (H.A.); raduly.lajos78@gmail.com (L.R.); zanoaga.oana@gmail.com (O.Z.)

**Keywords:** vinorelbine, NSCLC, EGFR, lncRNA, MAPK

## Abstract

Lung cancer remains the leading cause of cancer-related mortality worldwide, with non-small cell lung cancer (NSCLC) as the most common type. In addition, NSCLC has a high mortality rate and an overall adverse patient outcome. Although significant improvements have been made in therapeutic options, effectiveness is still limited in late stages, so the need for a better understanding of the genomics events underlying the current therapies is crucial to aid future drug development. Vinorelbine (VRB) is an anti-mitotic chemotherapy drug (third-generation vinca alkaloid) used to treat several malignancies, including NSCLC. However, despite its widespread clinical use, very little is known about VRB-associated genomic alterations in different subtypes of NSCLC. This article is an in vitro investigation of the cytotoxic effects of VRB on three different types of NSCLC cell lines, A549, Calu-6, and H1792, with a closer focus on post-treatment genetic alterations. Based on the obtained results, VRB cytotoxicity produces modifications on a cellular level, altering biological processes such as apoptosis, autophagy, cellular motility, cellular adhesion, and cell cycle, but also at a genomic level, dysregulating the expression of some coding genes, such as *EGFR*, and long non-coding RNAs (lncRNAs), including *CCAT1*, *CCAT2*, *GAS5*, *MALAT1*, *NEAT1*, *NORAD*, *XIST*, and *HOTAIR*, that are implicated in the mitogen-activated protein kinase (MAPK) signaling pathway. Therefore, although extensive validation is required, these results pave the way towards a better understanding of the cellular and genomic alterations underlying the cytotoxicity of VRB.

## 1. Introduction

Lung cancer constitutes the leading cause of cancer-related mortality globally, with an estimated number of deaths of 1.7 million, according to the Global Cancer Observatory (GLOBOCAN) [1]. Non-small cell lung cancer (NSCLC) is the most common form of lung cancer, constituting around 85% of all lung cancer cases [2]. As NSCLC is associated with a high mortality rate, even more so in advanced stages, many therapies have been developed for this condition [3]. Nonetheless, the literature suggests that the optimal outcomes achieved are through chemotherapy, often including combinations of platinum-based drugs and cytotoxic agents such as vinorelbine (VRB) [3].

Despite the developments and optimization of already commonly prescribed drugs, NSCLC has a low 5-year survival (less than 18% in late stages), often attributed to treatment resistance [4,5,6]. This resistance often arises from mutations in oncogenes, and, as such, identifying novel pathways for treatment resistance is essential to developing higher-efficacy therapies [5].

VRB is a *Vinca* alkaloid antimitotic that has been thoroughly studied as a single agent or combined with other treatments, with its efficacy proven in multiple cancers, including NSCLC [7,8,9,10,11,12]. VRB exerts its anticancer effects primarily by inhibiting microtubule dynamics, causing mitotic arrest and apoptosis [12,13]. Although VRB functions have been comprehensively assessed, studies evaluating its effects on novel oncogenes and pathways in which resistance to VRB occurs are been scarce [14]. Moreover, several articles have suggested tumor proliferation to take place during VRB treatment, with some suggesting a possible mechanism leading to epidermal growth factor receptor (EGFR) sensitization [15,16]. This suggests EGFR inhibitors in combination with VRB as a possible therapy for NSCLC.

Nonetheless, clinical trials provided conflicting results, with evidence that EGFR inhibition in combination with VRB does not improve survival in NSCLC [17,18]. Other research has indicated that *EGFR* mutations predict poor prognosis for NSCLC patients treated with VRB in combination with platinum-based chemotherapy, with evidence of variable treatment-response according to occurring co-mutations [19]. Previous preclinical studies have already shown that *EGFR* activity is altered following VRB treatment in breast cancer cells [20].

Further, research suggested possible involvement of long non-coding RNAs (lncRNAs) as prognostic signatures in lung cancer [21]. One review by Liu et al. suggested several lncRNAs as regulators of proliferation in lung tumors [22]. Multiple lncRNAs have been shown to have regulatory actions on the mitogen-activated protein kinase (MAPK) signaling pathway [23,24,25]. The MAPK pathway is a complex cascade of protein kinases that is known to play a pivotal role in the tumorigenesis, proliferation, apoptosis, and metastasis of cancer and other diseases [26]. However, no in vitro studies assessed the effects of VRB on lncRNAs relevant to the MAPK pathway in NSCLC so far.

The objectives of this study were to comprehensively assess the effects of VRB on 3 NSCLC cell lines in vitro, encompassing cellular functions such as viability, apoptosis, autophagy, and proliferation. Moreover, the objectives included assessing gene transcript expression alterations and correlations of *EGFR* and MAPK-associated lncRNAs.

To achieve these objectives, we first utilized MTT assays to determine the sensitivity of cell lines to various VRB doses. We also performed autophagy, apoptosis, and scratch assays to evaluate cell death within the cell cultures and assess the effects of VRB on apoptosis and cell migration parameters. Next, we performed cell cycle analysis to assess the antimitotic effects of VRB on cell lines. Finally, real-time polymerase chain reaction (qRT-PCR) was performed to assess the difference in expression between VRB-treated and control cell lines across a panel of 10 transcripts. To assess these expression differences, we performed statistical analysis and gene set enrichment analysis (GSEA).

Our qRT-PCR panel includes the tyrosine kinase receptor (TKR) encoding gene for epidermal growth factor receptor (*EGFR*) [27] as well as the following lncRNAs with proven involvement in MAPK pathway regulation: colon cancer-associated transcript-1 (*CCAT1*), colon cancer-associated transcript-2 (*CCAT2*), growth arrest-specific 5 (*GAS5*), HOX transcript antisense RNA (*HOTAIR*), HOXA distal transcript antisense RNA (*HOTTIP*), metastasis-associated lung adenocarcinoma transcript 1 (*MALAT1*), nuclear paraspeckle assembly transcript 1 (*NEAT1*), non-coding RNA activated by DNA damage (*NORAD*), and X inactive specific transcript (*XIST*) [28,29,30,31].

Here, we report the results of these assessments, which include novel findings regarding the variable responses of three NSCLC cell lines to VRB treatment. We further provide insights regarding possible mechanisms through which VRB induces paradoxical effects, potentiating tumor proliferation in NSCLC, depending on the genetic profile of the tumor. To our best knowledge, this is the first in vitro study to investigate this transcript panel utilized here using VRB on NSCLC cells.

## 2. Materials and Methods

### 2.1. Cell Lines and Treatment

Three human NSCLC cell lines, consisting of A549, Calu-6, and H1792, were maintained in Ham’s F12 Nutrient Mixture (Gibco^®^, Waltham, MA, USA, Cat. no. 11-765-054), Minimum Essential Medium (Gibco^®^, Cat. no. 11-095-080), and RMPI 1640 (Gibco^®^, Cat. no. 11-875-093), respectively. All media were supplemented with 10% fetal bovine serum (FBS, Gibco^®^, Cat. no. 16-629-525) and 1% Penicillin/Streptomycin, while the H1792 was additionally supplemented with 1% Glutamine. The culture medium and supplements were purchased from Sigma-Aldrich. The cell lines were grown in a humidified atmosphere at 37 °C and 5% CO_2_. We used VRB (GP8758, Glentham Live Sciences, Corsham, UK) and incubated the cells post-treatment for 48 h.

A549 (ATCC CCL-185) cells are reported to have been derived from lung carcinoma of epithelial-like morphology. Calu-6 cells (ATCC HTB-56) are derived from anaplastic lung carcinoma. The H1792 cell line (ATCC CRL-5895) is derived from lung adenocarcinoma and has epithelial morphology. Overall, all the cell lines come from metastatic NSCLC according to ATCC.

### 2.2. MTT Assay

The dose-dependent sensitivity of cancer cell lines to VRB was determined through the MTT assay. To begin, 1 × 10^4^ cells/well were cultured in 96-well culture plates for 24 h at 37 °C in 5% CO_2_ atmosphere incubators. After 24 h incubation, cell cultures were treated with different doses of VRB (1 nM, 5 nM, 10 nM, 25 nM, 50 nM, 75 nM, 100 nM, 250 nM). At 48 h after treatment, the medium was discarded, and 100 µL MTT solution was added to every well. After 2 h incubation at 37 °C, the MTT solution was removed, and the formazan crystal was solubilized with 100 µL DMSO (dimethyl sulfoxide). The absorbance was measured at 570/690 nm for the cell viability assay in a microplate reader (Synergy H1 Hybrid Reader Biotek, Winooski, VT, USA).

### 2.3. Apoptosis by Fluorescent Microscopy

We used the Multi-Parameter Apoptosis Kit (Cayman Chemicals, Ann Arbor, MI, USA, Cat. no. ab176750) for the fluorescence microscopy evaluation of apoptosis. Cell staining was performed according to the manufacturer’s protocol. Stained cells were analyzed at UV wavelength for Hoechst and 560/595 nm for Tetramethyl rhodamine ethyl ester (TMRE) staining on Olympus IX71 inverted microscope. Hoechst is a specific stain for the nucleus, while TMRE indicates the mitochondrial membrane activity potential.

In addition, to quantify the apoptotic effects associated with the VRB treatment, we used Caspase 3/7 and Hoechst staining according to the manufacturer’s protocol. We used a Nexcelom Celigo S Image Cytometer BFFL—AV for this process. Data analysis was performed with the dedicated software provided by the manufacturer.

### 2.4. Autophagy Assay

Using the Autophagy/Cytotoxicity Dual Staining Kit (Abcam, Cambridge, UK, Cat. no. ab133075), in line with the manufacturer’s recommended protocol, autophagy of the NSCLC untreated and VRB treated cell lines was assessed after 48 h incubation. Monodansylcadaverine (MDC) was used to detect autophagic vacuoles and Propidium iodide (PI) for dead cell identification. Autophagic cells were visualized by fluorescence microscopy.

### 2.5. Scratch Assay

Scratch assay was performed on all cell lines, using untreated and treatment groups. Cells were seeded in a 24-well plate, and treatment groups were given VRB IC_50_. The results were normalized at 0 h, 6 h, 24 h, and 48 h by brightfield microscopy.

### 2.6. Confluence

Confluence was determined following cell seeding in 6 well plates, separating each cell line into VRB treated and untreated. Measurement was then taken at 48 h, and visualization was performed using Nexcelom Celigo S Image Cytometer BFFL—AV.

### 2.7. Cell Cycle Analysis

Cell cycle analysis was performed using Nexcelom Celigo S Image Cytometer BFFL—AV to determine the cycle-arresting functions of VRB on all cell lines, with untreated cell lines used as control groups. Cells were marked by Propidium iodide (PI).

### 2.8. qRT-PCR

Following the assays and the determination of the VRB IC_50_ dose for the three NSCLC cell lines, qRT-PCR was performed for the *EGFR* gene, and *CCAT1*, *CCAT2*, *GAS5*, *HOTAIR*, *HOTTIP*, *MALAT1*, *NEAT1*, and *NORAD* lncRNA. Housekeeping (HK) genes used were *B2M*, *HPRT*, and *GAPDH*. RNA was first isolated from three untreated and three VRB-treated cells of each cell line using the Trizol (TriReagent Sigma-Aldrich, St. Louis, MO, USA) protocol, and the quantitative and qualitative control was performed by Nanodrop-1000 spectrophotometer (ThermoScientific, Waltham, MA, USA). cDNA synthesis was performed with a High-Capacity Reverse Transcription Kit (ThermoFisher Scientific, Waltham, MA, USA). qRT-PCR was subsequently performed on the predefined qRT-PCR panel using the SYBR^®^ Select Master Mix (Applied Biosystems, Waltham, MA, USA, Cat. no. 44-729-20) according to the manufacturer protocols. Threshold cycle (Ct) values were obtained through fluorescence emission. Ct values were normalized against the geometric mean of the three HK genes. The qRT-PCR data analysis was conducted using the ΔΔC_t_ method, as previously described by Berindan-Neagoe et al. [32].

### 2.9. Gene Set and Ontology Enrichment

GSEA and gene ontology (GO) analysis were performed in Python v3.11 [33] in anaconda within jupyter lab v3.6.3 [34,35], through the gseapy v1.0.6 [36] module. MSigDB gene sets were utilized for GSEA, found at https://www.gsea-msigdb.org/ (accessed on 20 September 2023). GSEA was performed on all available collections in the MSigDB database. Gene transcript ranking was performed by multiplying the average log2FC by −10 log of adjusted *p*-values. To adjust *p*-values, we used the Benjamini–Hochberg procedure. To adjust for the relatively small qRT-PCR panel, and the exploratory nature of this study, gene set matching size was set to 1. Other gene sets utilized were miRTarBase_2017 [37], and TRANSFAC and JASPAR PWMs to predict possible transcription factors and associated miRNAs [38].

### 2.10. Statistical Analysis

The non-parameteric *t*-test was utilized to determine significantly altered expression for the qRT-PCR panel, which was conducted using GraphPad Prism software v.9 (GraphPad Software, San Diego, CA, USA). To determine inter-cell-line significantly altered expression, the ANOVA one-way test was performed on ΔΔC_t_ values; this was performed using numpy v1.23.5 [39] and scipy v1.10.1 [40] modules in python [33]. Values for lncRNA/gene expression are represented as mean ± standard deviation. For all the statistical analyses performed, a *p*-value < 0.05 was considered statistically significant. For the ANOVA test, to denote the statistical significance for inter-cell-line expression difference, we used the Bonferroni corrected *p*-value < 0.008.

For Pearson’s correlation coefficients determination, numpy v1.23.5 [39] and scipy v1.10.1 [40] modules were used in python [33]. Visualization of Pearson’s correlation coefficients, the differentially expressed transcripts, as well as GSEA results was conducted through the python [33] modules matplotlib v3.7.1 [41], seaborn v0.11.2 [42], and gseapy [36]. Datasets were prepared through the pandas v1.5.3 [43] module.

## 3. Results

### 3.1. VRB Induces NSCLC Cell Death at Different IC_50_ Depending on the Cell Line

**Figure 1:**
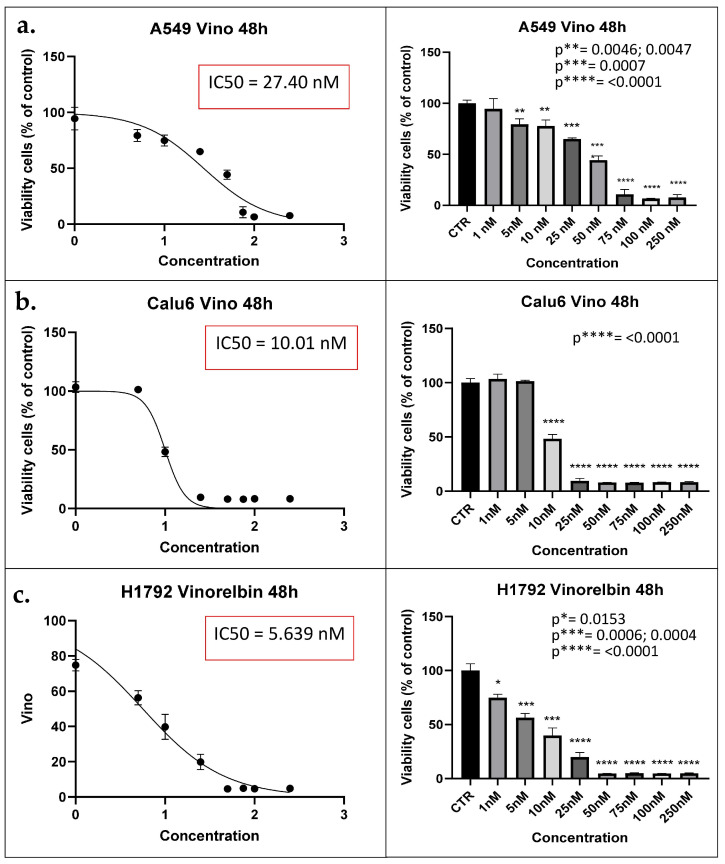
MTT assay results denote IC_50_ for each cell line. (**a**) A549 cell lines viability reaches 50% the original population at 27.40 nM at 48 h (**left**). Concentration distribution and efficacy of VRB on A549 cell lines (**right**). (**b**) Calu-6 cell lines viability decreased to half the original population at IC_50_ of 10.01 nM (left). Concentration is shown to be producing inhibitory effects at 10 nM (*p* < 0.0001) (right). (**c**) H1792 cell lines decrease in population according to concentration (**left**). H1792 VRB treated cell population decreased to half at 5.639 nM of VRB (**right**). IC_50_: Half maximal inhibitory concentration; CTR: Controls (Untreated). Vino: VRB (treated).

Each of the treated NSCLC cell lines was exposed to different VRB concentrations (1 nM, 5 nM, 10 nM, 25 nM, 50 nM, 75 nM, 100 nM, and 250 nM) to determine the IC_50_. Readings for the MTT assays at 48 h revealed that the IC_50_ for the A549, Calu-6, and H1792 treatment groups were 27.40 nM, 10.01 nM, and 5.639 nM, respectively (*p* ≤ 0.0001 for all) (as shown in Figure 1). Following the determination of IC_50_ for each cell line, the same concentration was utilized in both the control and treated cell populations from all cell lines for the subsequent experiments.

### 3.2. VRB Confers Cell Death via Apoptosis with Variable Efficacy

**Figure 2:**
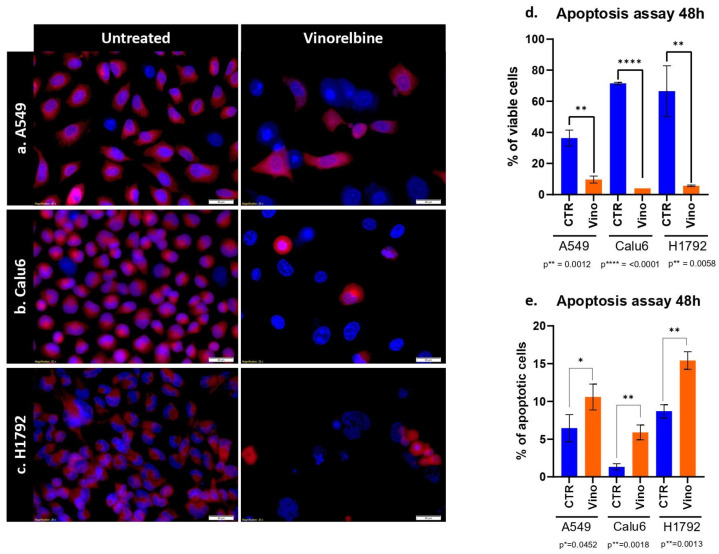
Apoptosis assay of VRB on NSCLC cell lines. (**a**) Comparison between untreated A549 cell lines and VRB-treated cell lines. (**b**) Comparison between untreated Calu-6 cells and VRB-treated cells. (**c**) Demonstrated a comparison between control H1792 cells and VRB-treated cells. Red fluorescence indicates membrane mitochondrial activity, and blue indicates nuclear activity. VRB-treated cells undergo apoptosis and lose mitochondrial activity, and therefore decrease in number. (**d**) Apoptosis assay showing an increase in apoptotic cells % in VRB treated populations in variable degrees compared to controls. (**e**) Analysis of viable cell lines shows higher VRB-untreated cell populations than VRB-treated cells. CTR: Controls (untreated). Vino: Vinorelbine (treated).

Results for the apoptosis assay at 48 h showed a significant decrease in the population of all cell lines, with variable efficacy. As the most significant decrease in cell line population was observed in Calu-6 cell lines (*p ≤* 0.0001) (Figure 2). Further analysis revealed that the most significant prevalence of apoptotic cells was among the H1792 cell lines (*p* = 0.0013).

### 3.3. VRB Induces Autophagocytosis in All NSCLC Cell Lines

**Figure 3:**
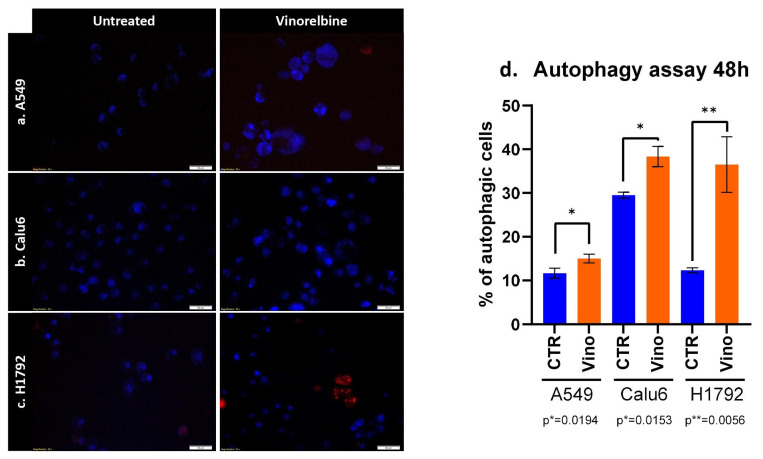
Autophagy induced by VRB on (**a**) A549, (**b**) Calu-6, and (**c**) H1792 cell lines denotes alterations in autophagosomes following VRB treatment for each cell line. (**d**) The percentage of autophagic cells after VRB treatment and 48 h or incubation.

As for the autophagy assays, similar results were obtained in terms of the significant efficacy of VRB in causing autophagocytosis amongst all cell lines. However, the most significant difference between the treated cell lines and controls was observed in the H1792 cell line, with the lowest significance observed in the A549 cell line (*p* = 0.019) (Figure 3).

### 3.4. NSCLC Cells’ Migration Is Altered under VRB Treatment

**Figure 4:**
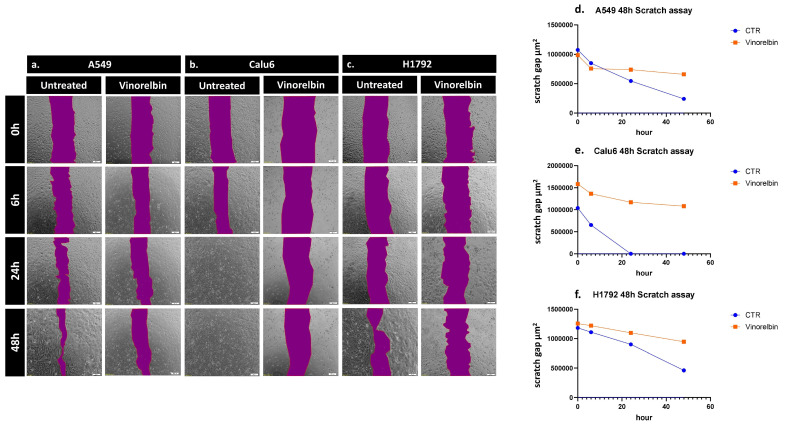
Scratch assay results in NSCLC cells. Change in the healing rate of (**a**) A549, (**b**) Calu-6, and (**c**) H1792 cell lines, observed by microscopy. (**d**) Analysis of VRB-treated A549 cells healing rate in comparison to controls. (**e**) Analysis of VRB-treated Calu-6 cells healing rate in comparison to controls. (**f**) Analysis of VRB-treated H1792 cells healing rate in comparison to controls.

To further assess the variation in the in vitro healing rate and migration across the NSCLC cell lines following VRB treatment, scratch assay was performed. The scratch assay revealed that Calu-6 controls had the fastest healing rate, completely returning to baseline cell population numbers at 24 h while simultaneously being the most affected by VRB treatment. VRB-treated Calu-6 cell lines had the largest scratch gap difference amongst the cell lines, signifying they had the most altered motility under VRB treatment. H1792 cell lines seemed to be least affected by VRB amongst the cell lines, compared to controls, albeit still affected. As for A549 cells, control cell populations seemed to be exponentially recovering, while VRB-treated cells appeared to have drastically decreased in wound healing rate from around 6 h until the last reading at 48 h (Figure 4).

### 3.5. VRB-Treated Cell Lines Had Significantly Altered Confluence in Comparison to Untreated Cells

**Figure 5:**
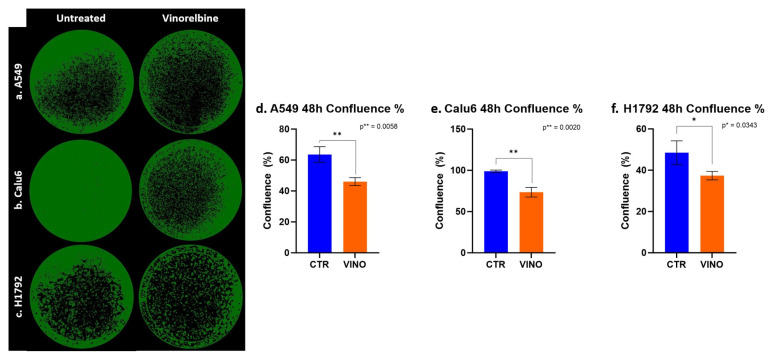
NSCLC cells colony forming inhibiting effects exerted by VRB. (**a**) A549 cell lines confluence decreased after VRB introduction. (**b**) Calu-6 cell line confluence was impacted significantly after VRB treatment. (**c**) H1792 cell confluence was affected the least by VRB, although the results from this cell line remained significant. (**d**–**f**) represent the difference in confluence and colony-forming capacity in control and VRB-treated cells.

Confluence was also assessed, further demonstrating the significant efficacy of VRB across all cell lines, with the most significant difference between untreated and treated cell lines at 48 h in Calu-6 cells (*p* = 0.002). With less efficacy appearing on H1792 cells while still achieving statistical significance (*p* = 0.0343) (Figure 5).

### 3.6. VRB Confers Cell Cycle Arrest Differently in a Cell-Line-Dependent Manner

**Figure 6:**
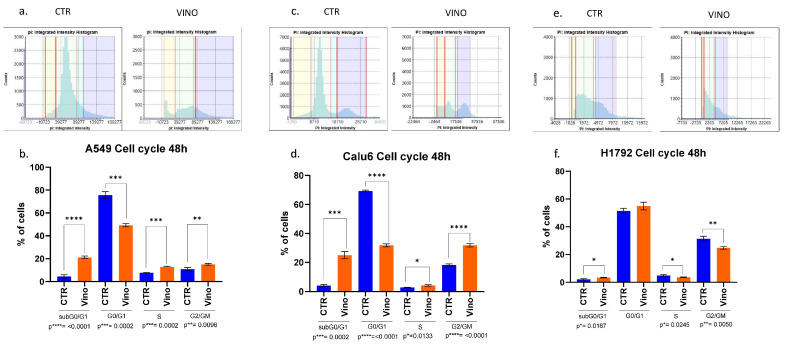
Cell cycle analysis results. The amount of PI fluorescent intensity is correlated to the amount of DNA within each cell. Since the amount of DNA doubles (2n → 4n) between the G1 and G2 phases, the cell population’s fluorescent intensity doubles from 2000 to 4000 fluorescence units. (**a**,**b**) PI intensity histogram and cell cycles populations analysis at 48 h for A549 cell lines, respectively. A549 VRB treated cell line had a higher population of cells in the subG0/G1 phase than controls. The opposite happened in the G0/G1 phase, indicating more cells might have been arrested in the subG0/G1 phase following VRB treatment. Other significant differences were observed in different cell cycle phases comparing VRB-treated cells to controls. (**c**,**d**) PI histogram and cell cycle analysis for Calu-6 cell lines. Similarly, as in the A549 cells, VRB-treated cells had a higher population in the subG0/G1 phase than controls, leading to a decreased population in the G0/G1 phase. Following similar patterns to A549 cells in the subsequent phases, albeit to a higher degree. (**e**,**f**) for H1792 cells, VRB-treated cells had a higher population than control cells in the subG0/G1 phase. However, in the G0/G1 phase, there was no significant difference in the VRB-treated cells and controls. Nonetheless, significantly fewer cells were present in the S and G2/GM cell cycle phases in the VRB-treated cells than controls. CTR: Untreated cell lines. Vino: VRB treated cells.

Since the NSCLC cell lines showed variable treatment responses to VRB, we sought to further understand whether VRB affects cell cycles depending on the cell line. Cell cycle analysis showed significant differences in all VRB-treated cell populations compared to controls at all cell cycles across the board, with one exception observed in H1792 cells in the G0/G1 transition phase. Although not achieving significant differences in all cell cycles in H1792, VRB seemed to have some effects on the cell line, arresting other cell cycle phases despite demonstrating the lowest efficacy compared to the other cell lines (Figure 6).

VRB showed variable efficacy in arresting cells in different cell cycle phases. For example, A549 cells sustained the most significant effects on the subG0/G1 inactive phase (*p* ≤ 0.0001) while experiencing less effects on the G2/GM and S phases, respectively. Nevertheless, the results remained significant. VRB demonstrated similarly substantial effects in both the G0/G1 and G2/GM phases on the Calu-6 cells (*p* ≤ 0.0001). At the same time, they incurred the most minor efficacy in their S phase (*p* = 0.013). Table 1 compares pre- and post-treatment cell populations in each phase of the cell cycle.

Overall, VRB had reliable efficacy on all cell lines, with various effects on different aspects of the selected NSCLC cell lines. VRB-treated cells were significantly more apoptotic, produced more autophagosomes, had less colony-forming capacities, and demonstrated a weaker recovery rate than control cell lines. VRB-treated cells showed substantially more inhibited cell proliferation through the antimitotic effects of VRB, indicating significant cell cycle differences compared to controls across all cell lines except for the H1792 cells.

### 3.7. Changes in EGFR and lncRNA Expression Profiles after VRB Treatment Are Cell Line-Dependent

Cell lines were divided into six samples, three controls and three VRB-treated groups, using the VRB IC_50_ for each cell line as previously described. Following the extraction of cDNA from the cell lines and sequencing of the preselected qRT-PCR panel described in the methodology, a significant difference was found in the relative expression of several gene transcripts. Statistical analysis of the relative expression levels in VRB-treated versus untreated cells was conducted using the non-parametric *T*-test. *CCAT1* and *CCAT2* expression levels were undetermined in Calu-6 and H1792 cell lines. *NEAT1* and *HOTAIR* levels were undetermined in Calu-6. *HOTTIP* levels were undetermined in all cell lines.

**Figure 7:**
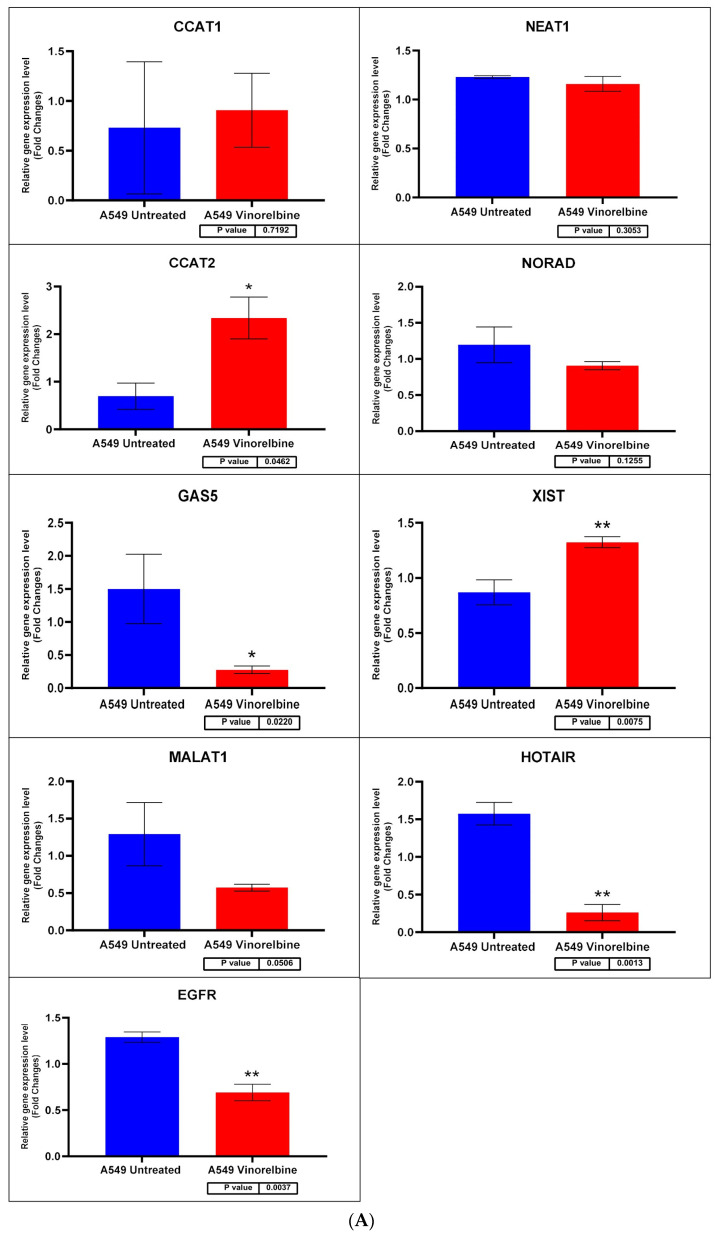

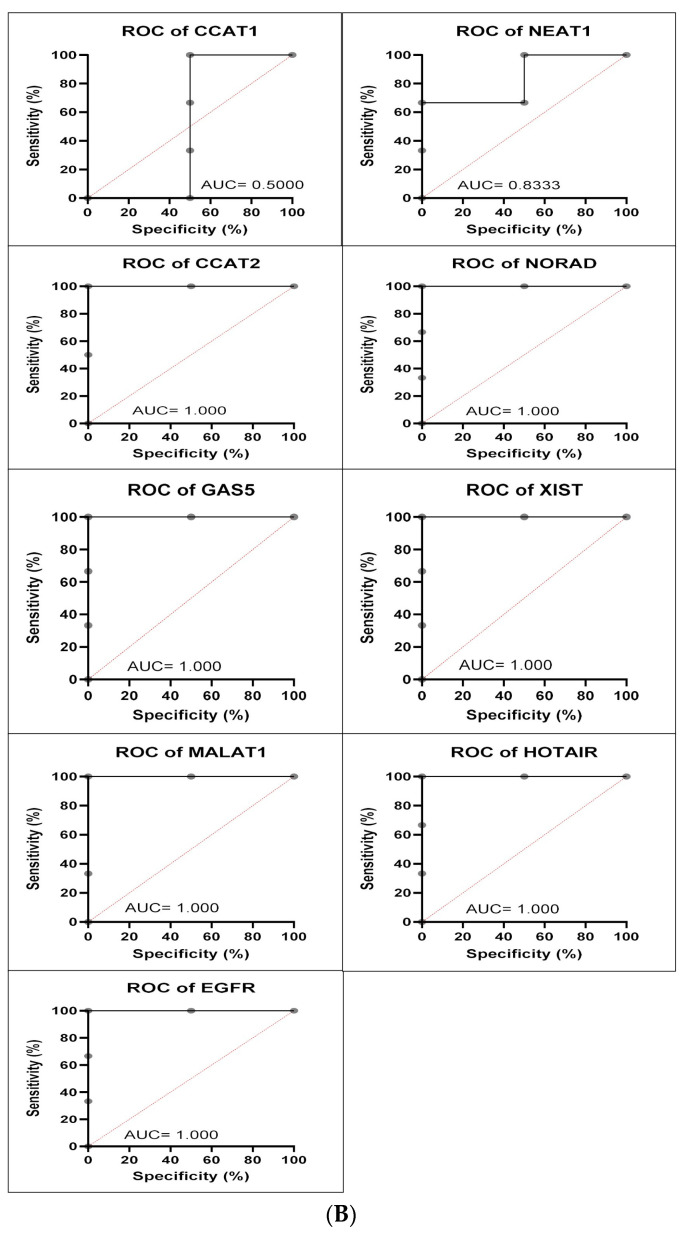
(**A**) A549 cell line fold change for the qRT-PCR results. Differential expression levels between VRB treated and untreated A549 cells which could be determined for the EGFR gene and CCAT1, NEAT1, CCAT2, NORAD, GAS5, XIST, MALAT1, and HOTAIR lncRNAs (* *p* < 0.05, ** *p* < 0.01). (**B**) ROC curves measuring the sensitivity and specificity percentage of the differential expression levels from the VRB-treated and untreated A549 cells which could be determined for the *EGFR* gene and *CCAT1*, *NEAT1*, *CCAT2*, *NORAD*, *GAS5*, *XIST*, *MALAT1*, and *HOTAIR* lncRNAs. AUC = Area Under the Curve.

**Figure 8:**
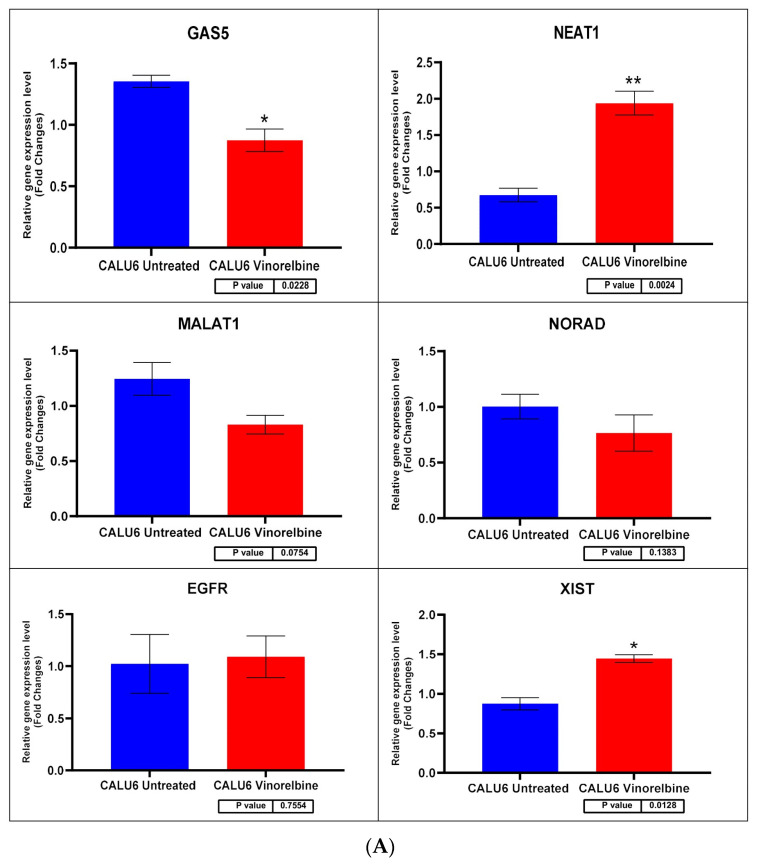

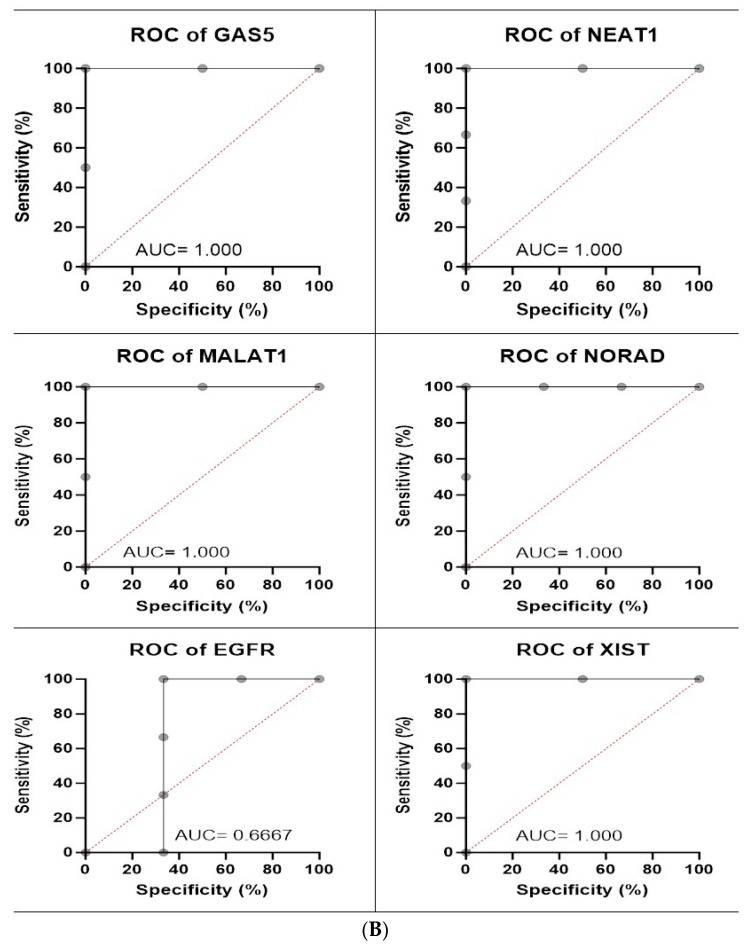
(**A**) Calu-6 cell line fold change for the qRT-PCR results. Differential expression levels between VRB-treated and untreated Calu-6 cells which could be determined for the *EGFR* gene and *GAS5*, *NEAT1*, *MALAT1*, *NORAD*, and *XIST* lncRNAs (* *p* < 0.05, ** *p* < 0.01). (**B**) ROC curves measuring the sensitivity and specificity percentage of the differential expression levels from the VRB-treated and untreated Calu-6 cells which could be determined for the *EGFR* gene and *GAS5, NEAT1, MALAT1, NORAD*, and *XIST* lncRNAs. AUC = Area Under the Curve.

**Figure 9:**
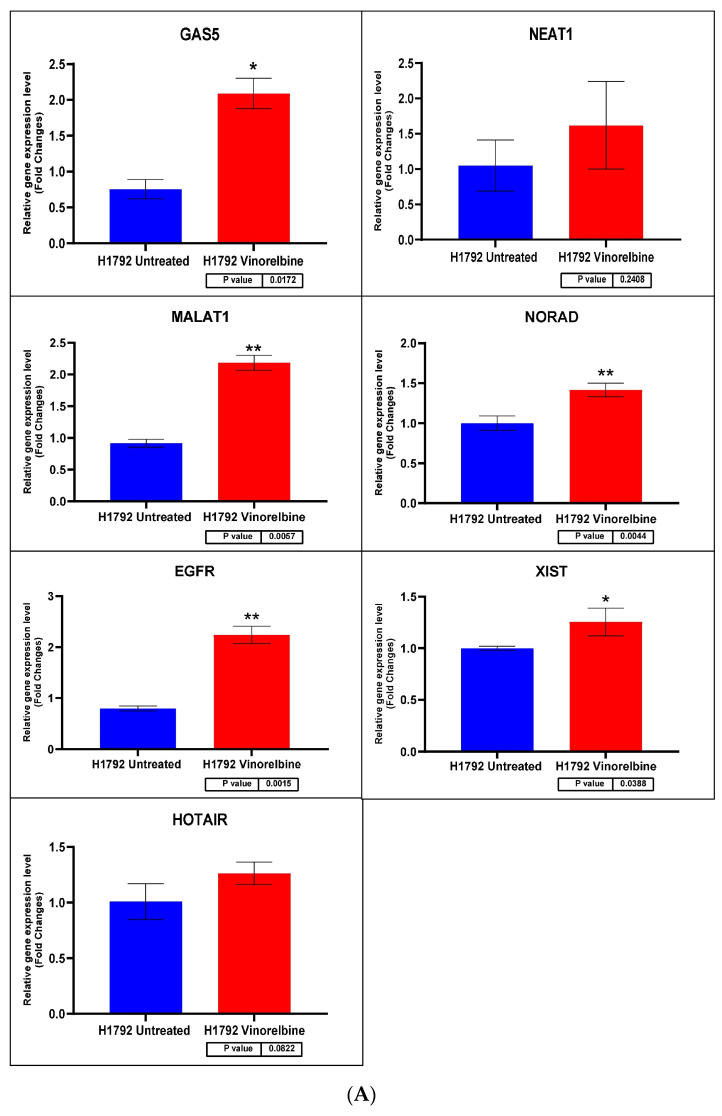

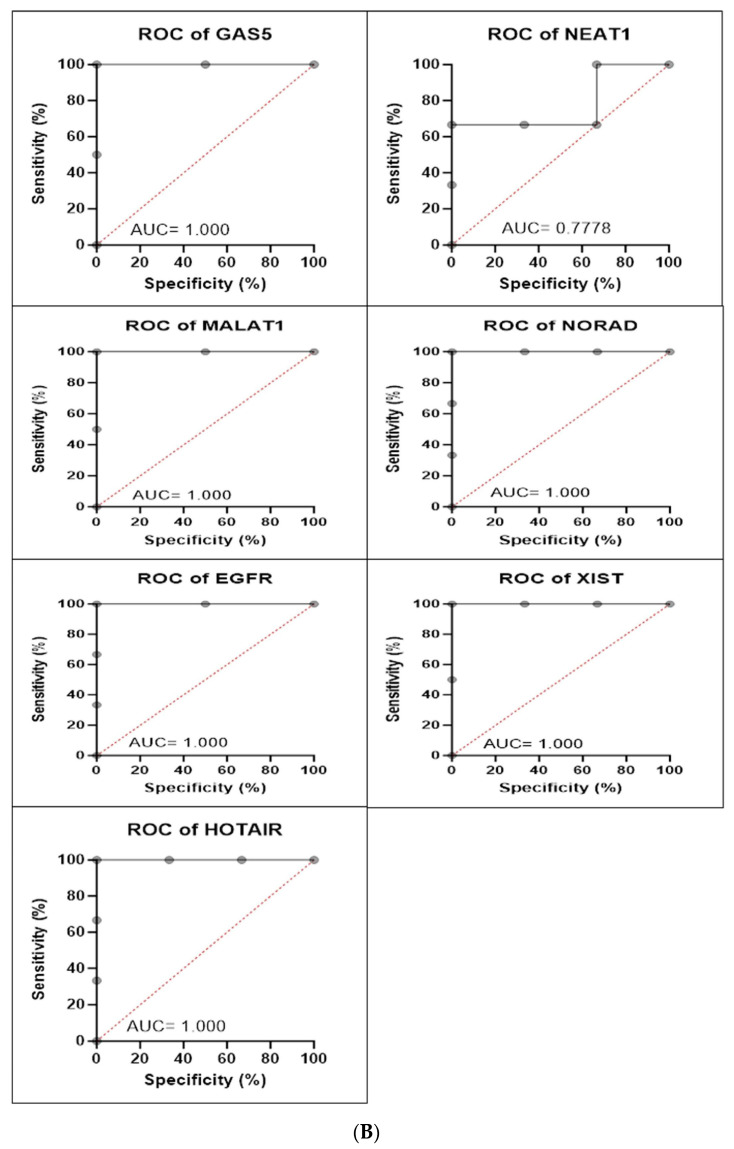
(**A**) H1792 cell line fold change for the qRT-PCR results. Differential expression levels between VRB-treated and untreated H1792 cells which could be determined for the *EGFR* gene and *GAS5*, *NEAT1*, *MALAT1*, *NORAD*, *XIST*, and *HOTAIR* lncRNAs (* *p* < 0.05, ** *p* < 0.01). (**B**) ROC curves measuring the sensitivity and specificity percentage of the differential expression levels from the VRB-treated and untreated H1792 cells which could be determined for the *EGFR* gene and *GAS5, NEAT1*, *MALAT1*, *NORAD*, *XIST*, and *HOTAIR* lncRNAs. AUC = Area Under the Curve.

Regarding transcripts with significantly altered expression, *CCAT2* had higher expression levels in VRB-treated A549 than controls (Figure 7 shows the qRT-PCR results for A549 cells). *GAS5* had less expression in A549 and Calu-6 cells but higher expression in H1792 cells in comparison to control cells (qRT-PCR results for Calu-6 cell line shown in Figure 8). *MALAT1* had significantly higher relative expression only in H1792 cell lines (Figure 9 shows expression analysis results for H1792 cells). As for *EGFR*, it had lower expression in A549 cell lines but higher expression in H1792 cells in comparison to controls. *NORAD* had significantly higher expression in H1792 cells while decreased expression in other VRB-treated cells, although not reaching statistical significance. *XIST* expression was increased across all VRB-treated cell lines. Whereas for *HOTAIR*, VRB-treated A549 cells had significantly less expression of this lncRNA. However, H1792 cells had a higher expression than control cell populations, although it did not reach the significance threshold.

### 3.8. Statistical Analysis Reveals Altered Correlations of Expression Profiles in VRB-Treated NSCLC Cell Lines

**Figure 10:**
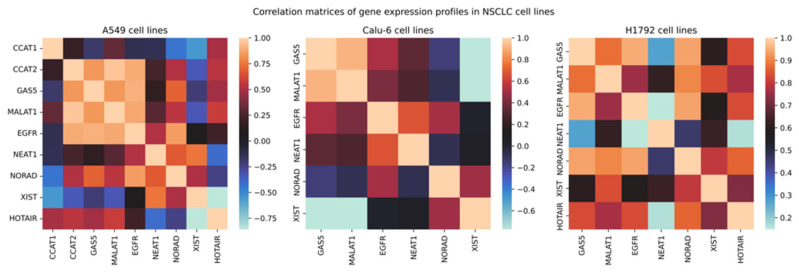
Correlation matrices of relative expression. Legend corresponds to Pearson’s correlation coefficient.

Statistical analysis of correlations of relative expression revealed several relationships. Interestingly, *GAS5* had a Pearson’s correlation coefficient of 0.84, 0.83, 0.9, and 0.7 with *CCAT2*, *MALAT1*, *EGFR*, and *NORAD,* respectively, in A549 cell lines (Figure 10). As for the Calu-6 cell line, *GAS5* demonstrated a correlation of −0.77, 0.53, and 0.91 with *XIST*, *EGFR*, and *MALAT1,* respectively. In H1792 cell lines, *MALAT1* showed a Pearson’s coefficient of 0.77, 0.84, 0.91, 0.76, and 0.88 with *GAS5*, *XIST*, *NORAD*, *EGFR*, and *GAS5*, respectively. Interestingly, *MALAT1* showed consistently high correlations with *GAS5* amongst all cell lines. Due to the change of the relative expression in multiple cell lines, an inter-sample ANOVA one-way test was performed in both controls and treated cell line populations separately to determine significance between cell lines in relative expression, applying Bonferroni’s corrected *p*-value (*p* < 0.008). Statistical significance was observed in relative expression across all VRB-treated cell lines in *GAS5*, *MALAT1*, and *EGFR*.

**Figure 11:**
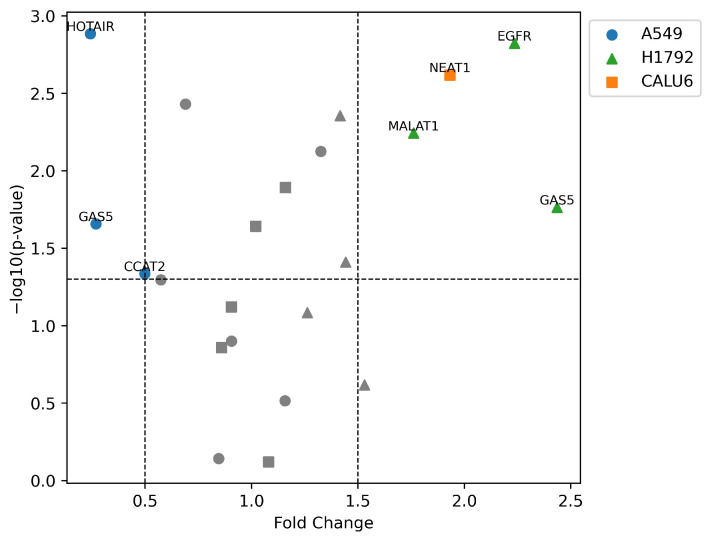
Volcano plot of differentially expressed transcripts in NSCLC cell lines. –log_10_(*p*-value) as a significance threshold was set to 1.3, equal to *p*-value = 0.05. Higher –log_10_(*p*-value) indicates higher significance of expression alteration. Significant fold change was considered below 0.5 (50% downregulation) or above 1.5 (150% upregulation). Shapes of data points indicate the cell line of the corresponding lncRNA/gene.

Statistically significant expression differences were not observed in many transcripts in the VRB-treated cell lines, with some values being undetermined. Nonetheless, analysis of ΔΔ*C*_t_ changes was performed in all cell lines, documenting possible correlations in the available data (Figure 11 shows a volcano plot of significantly upregulated and downregulated transcripts).

### 3.9. GSEA and GO Analysis Show Cell-Line-Dependent Mechanisms of VRB Treatment Responses

Ranking the qRT-PCR panel according to the methodology was performed to further investigate inter-sample differences in gene expression. Subsequently, GSEA was conducted to further understand the pathways affected by VRB and the functionality of the upregulated and downregulated transcripts in our panel.

**Figure 12:**
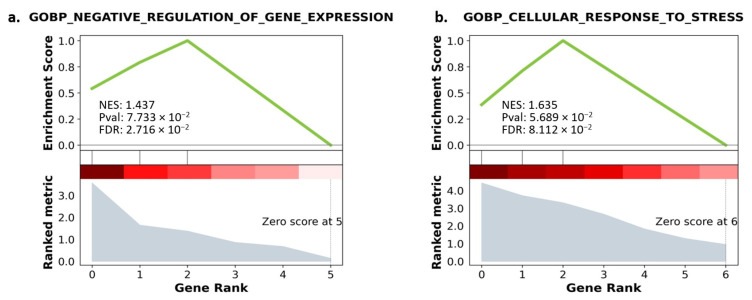
(**a**) GO enrichment with gene ranking in Calu-6. (**b**) GO enrichment with gene ranking in H1792. Lower gene ranking refers to upregulated genes, while higher gene ranking refers to downregulated genes. The ranking metric explains the degree of upregulation and downregulation. The enrichment score explains the matched genes’ involvement in the enriched pathway. NES: Negative enrichment score. Pval: *p*-value before adjustment. FDR: False discovery rate.

As our transcript panel was relatively small, and the data regarding their functions were scarce, the minimum size for gene set enrichment was set to 1. Due to the low statistical power, a false discovery rate (FDR) ≤ 0.1 was considered a significant enrichment of the gene sets. Surprisingly, no gene sets were enriched with our qRT-PCR panel from the A549 cell line besides for the transcription factor ETS1. However, two biological processes were significantly enriched for the other cell lines (Figure 12a,b).

Results of GO analysis for the Calu-6 cells revealed that *NEAT1*, *XIST*, and *GAS5* were enriched in negative regulation of gene expression. For the H1792 cells, a significantly enriched ontology biological process was relevant to the cellular response to stress, enriched with *EGFR, GAS5*, and *MALAT1*. Downregulated transcripts from the H1792 cell lines further enriched other biological processes involved in miRNA metabolic processes, although with a higher FDR than our predefined threshold (FDR = 0.106) (Table 2).

## 4. Discussion

Although the overall survival rate of NSCLC patients has improved during the past years, the genetic alterations NSCLC presents and its inter-patient heterogeneity represent major obstacles to effective treatments [49]. This genetic heterogeneity has been implicated in the variable responses to VRB treatment in NSCLC patients, as both single agent and in combination with other drugs [19]. This variation in tumor response to treatment thereof necessitates further investigations.

This article provides insight into many aspects of both the multifaceted effects of VRB on NSCLC cell lines and the functions exerted by the predefined qRT-PCR panel, particularly in this condition. Moreover, this is the first study to investigate VRB’s effects on this specific set of lncRNAs in NSCLC cell lines in vitro, in particular *CCAT1*, *CCAT2*, *MALAT1*, *NEAT1*, *GAS5*, *NORAD*, *XIST*, *HOTAIR*, and *HOTTIP*.

Interestingly during our experiments, amongst all NSCLC cell lines, there were different responses to the applied VRB concentrations. The A549 cell line required 27.40 nM of VRB to reach 50% of its original cell population, while H1792 cells required only 5.639 nM of VRB to reach half the population. Further, Calu-6 cells required 10.01 nM. This is likely due to the variable sensitivity of the NSCLC cell lines to VRB [50].

Further assessing VRB effects, using the IC_50_ concentration for each cell line conferred significant apoptosis on all cell lines with variable efficacy. VRB also induced autophagocytosis and reduced the scratch-healing rate effectively in all cell lines, while further demonstrating variable efficacy across the NSCLC cells. The results here are in line with previous results from our lab and literature [51,52,53]. VRB has been well documented to produce variable effects in cancer therapy, owing to both, the type of tumor and its respective genetic profile, and the administration pattern of the drug [54,55,56].

The effects of VRB on the cell cycle in the NSCLC cell lines were particularly interesting. Our results show that VRB-treated A549 and Calu-6 cells had a higher population in the G2/M phases of the cell cycles in comparison to controls, contrary to H1792 cell line. This discrepancy may be attributed to the complex range of effects in which VRB affects cell lines, and that it may not cause apoptosis in NSCLC explicitly through mitotic arrest [57]. Moreover, it could be possible that prior to VRB treatment, the cells within each cell line were undergoing different stages of differentiation. Literature has previously suggested that VRB affects cell cycle differently according to the phase during which the cell is treated, and the responses are always cell line-dependent [58].

Although it has been established that VRB exerts its antitumor effects through altering mitotic spindle dynamics in a dose-dependent manner, by binding to beta-tubulin at the ends of microtubules, the precise pathways that cause its effects are yet to be fully characterized [12,13,53,54,59]. This is in part due to evidence showing that VRB is capable of conferring apoptosis in colorectal cancer cell lines regardless of mitotic arrest [57].

The effects of VRB on the cell cycle should be further characterized by future research in vivo, as it may be possible that cells that are able to survive VRB actions may have enhanced proliferation as they accumulate in the G2/M phases of the cell cycle following DNA repair. Research provided evidence that mitotic arrest can be overcome through the depletion of several cellular proteins [60,61]. Further, an investigation by Busacca et al. revealed that *BRCA1* silencing revoked VRB-induced cell cycle arrest in mesothelioma [62].

To further understand the effects of VRB, several transcripts were analyzed through qRT-PCR and returned several results. Inter-cell-line differences in expression were observed in *EGFR*, *GAS5*, and *MALAT1* through the ANOVA test. Comparing controls and VRB-treated cells in each cell line using the *t*-test revealed that the alteration of *EGFR* expression was cell line-dependent. *EGFR* was downregulated in A549 yet upregulated in H1792. Similarly, *GAS5* followed the same expression patterns. The literature suggests that *GAS5* overexpression is associated with suppressed cell growth and proliferation in multiple cancers, and it is considered a sign of chemosensitivity [63,64,65]. *GAS5* has been further shown to have a negative correlation with *EGFR* in treatment-naïve NSCLC tissues [66].

Surprisingly, VRB downregulated *GAS5* in the A549 cells, and we have observed a Pearson’s correlation coefficient of 0.9 and 0.95 between *GAS5* and *EGFR*, in A549 and H1792 cells respectively. GO analysis indicates that the increase in both *GAS5* and *EGFR* expression in H1792, is likely due to a process involved in the cellular response to nitrogen compounds, which is VRB in this case (GO:1901698). Enriched gene sets with *GAS5* and *NEAT1* in Calu-6 suggest that the significant upregulation of *GAS5* and *NEAT1* had to do with functional responses to the drug, indicating a possible mechanism of interaction, likely through the transcription factor AP-2 alpha [31,67].

Literature had previously reported that the cell lines included in our experiment have different genetic profiles [68]. Moreover, reports indicated that VRB-treated cells with mutated *EGFR* had increased expression of the receptor, leading to increased sensitivity to *EGFR* Tyrosine Kinase Inhibitors (TKIs) [15,20,69]. These reports could possibly explain our observation of the variability in *EGFR* expression in A549 and H1792 VRB-treated cells. This variable response to VRB could have many implications in a clinical setting, possibly explaining resistance development in some populations and decreased resistance in others [15,20,69]. As some studies and clinical trials have confirmed combinations of VRB and *EGFR* TKIs have high efficacy, further investigation of the relationship between *EGFR* and VRB is warranted [16,70,71,72,73].

The positive correlation between *GAS5* and *EGFR* under VRB treatment requires further attention. As *GAS5* downregulation is associated with poor prognosis in NSCLC, the effects of VRB could potentially aggravate the tumor and induce further metastasis [74,75]. Clinically, *GAS5* was suggested to have value as a diagnostic biomarker and as a therapeutic target in several cancers as well [76,77]. Downregulation of *GAS5* lncRNA was associated with TKI resistance previously, which underlies the importance of investigating this particular lncRNA under VRB treatment [78]. The clinical significance of *GAS5* expression alteration under VRB treatment, is that it may render NSCLC cell subpopulations within the same tumor resistant towards VRB under repetitive exposure. Future research should target *GAS5* expression in vivo under repetitive VRB administration.

Moreover, VRB increased the expression of *XIST* over all cell lines. Upregulated *XIST*, *MALAT1*, and *NEAT1* lncRNAs were predicted to modulate and promote cell development and further metastasis in multiple cancers through mir-124-3p, but no studies in lung cancer were done regarding other functions of these lncRNAs, or their response to VRB [79,80]. Overexpression of these lncRNAs and their variable responses to VRB warrants further research, as they could explain possible pathways in which resistance to VRB or further metastasis occurs or predict poor response or prognosis in certain NSCLC patients. It is possible that targeting these lncRNAs in combination with VRB could improve survival rate.

Other interesting associations included the consistently high correlation between *MALAT1* and *GAS5* across all cell lines. These lncRNAs, in addition to *XIST*, were hypothesized to play a role in radiosensitivity in A549 cells [81]. Nonetheless, studies regarding their interplay, and specifically the interactions between *GAS5* and *MALAT1*, are lacking, which warrants future investigation.

Overall, while VRB has reliable efficacy and had undergone thorough assessment, it may have possible interactions with genetic pathways that could contribute to increased tumorigenesis [82]. Therefore, clinical setting should take into consideration the genetic profile of the NSCLC tumor before prescribing *Vinca* alkaloids as they may contribute to worsened prognosis due to lncRNAs that increase chemoresistance or contribute to the metastasis and proliferation of the primary tumors. Also, based on our results, *EGFR* may also be implicated in VRB response, especially when correlated with other MAPK-associated lncRNAs.

Nonetheless, our study has several limitations. Our sample replicates were of relatively low number, as well as our preselected gene panel, leading to low statistical power and a risk of bias. We also considered FDR < 0.1 to be significant enrichment of gene sets. However, as these lncRNAs, in addition to *EGFR*, act on similar pathways relevant to MAPK, that made it possible to investigate multiple aspects of the pathway under the effects of VRB. Therefore, we recommend future research to further investigate the possible interactions of *Vinca* alkaloids with these lncRNAs in larger studies as well as in clinical settings. The utility of MAPK-associated lncRNAs may be valuable as therapeutic targets that could possibly significantly improve patients’ survival.

## 5. Conclusions

Our data indicate that despite the reliable efficacy of VRB, it might have possible unexplored interactions with *EGFR* and MAPK-associated lncRNAs that could increase the likelihood of tumor proliferation and metastasis. Relationships that were particularly interesting included those with *GAS5*, *XIST*, and *EGFR*. The consistent correlation of *MALAT1* and *GAS5* across all NSCLC cell lines indicates a possible mechanism of interaction. Results suggest that *Vinca* alkaloids have several interactions with MAPK pathway regulatory genes, depending on the genetic profile of the tumor.

As our replicate size was of a low number, and our qRT-PCR panel was small limiting the statistical power of our study, the conclusions of this research should be interpreted with caution. Therefore, we recommend future research to further investigate these possible interactions in larger samples and in clinical trials, as targeting these pathways could lead to improved prognosis and higher efficacy in TKI-resistant populations.

## Figures and Tables

**Table 1:** Pre- and post-treatment comparison of the cells number percentage in each phase of the cell cycle.

Cell Line	Mean Number of Cells (%)							
	subG0/G1	G0/G1	S	G2/M	subG0/G1	G0/G1	S	G2/M
	Untreated Cells (Control)				Cells Treated with VRB			
A549	4.49	75.59	7.68	10.9	21.15	49.28	12.77	15.16
Calu-6	4.11	69.31	2.72	18.31	25.06	31.85	4.19	31.84
H1792	2.09	51.34	4.86	31.36	3.36	5.49	3.56	24.75

**Table 2:** Gene ontology enrichment analysis for A549, Calu-6, and H1792 cell lines. **↓** indicates downregulation of the matched gene in the enriched term/gene set. **↑** indicates upregulation. FDR: False discovery rate.

Source	Term	Definition	FDR	Matched Genes	Cell Line
TRANSFAC and JASPAR PWMs	TFAP2A (human)	Transcription factor AP-2 alpha (activating enhancer binding protein 2 alpha). This gene encodes a transcription factor with complex functions, and defects have been observed in several diseases [44]. Was observed to potentiate lung adenocarcinoma [45].	0.015023	*HOTAIR* ↓ *NEAT1* ↓	H1792
GO:0010629	GOBP_NEGATIVE_REGULATION_OF_GENE_EXPRESSION	Describes any process that reduces frequency rate or extent of gene expression.	0.027157	*NEAT1* ↑ *XIST* ↑ *GAS5* ↑	Calu-6
MiRTarBase_2017	hsa-miR-124-3p	lncRNA with post-transcriptional regulatory functions of gene expression. Suppresses metastasis and defects are associated with several diseases including breast and lung cancer [46].	0.036153	*HOTAIR* ↓ *NEAT1* ↓	H1792
TRANSFAC and JASPAR PWMs	ETS1 (human)	Proto-oncogene with complex functions, could induce metastasis in NSCLC [47].	0.039859	*HOTAIR* ↓ *GAS5* ↓	A549
MiRTarBase_2017	hsa-miR-21-5p	Was found to be upregulated in most human cancers, considered to predict prognosis of lung cancer [48]	0.076526	*EGFR* ↑ *GAS5* ↑	H1792
GO:0033554	GOBP_CELLULAR_RESPONSE_TO_STRESS	Describes processes that alters cellular activity in terms of gene expression or enzyme production or similar functions.	0.081121	*EGFR* ↑ *GAS5* ↑ *MALAT1* ↑	H1792
GO:2000628	GOBP_REGULATION_OF_MIRNA_METABOLIC_PROCESS	Describes processes that regulate frequency, rate, or extent of miRNA metabolic process.	0.10689	*EGFR* ↑ *GAS5* ↑	H1792
GO:0062197	GOBP_CELLULAR_RESPONSE_TO_CHEMICAL_STRESS	Processes altering cellular activity due to the induction of a stress response as a result of a chemical stimulus.	0.10689	*EGFR* ↑ *GAS5* ↑	H1792
GO:0034599	GOBP_CELLULAR_RESPONSE_TO_OXIDATIVE_STRESS	Processes that produce alterations in cellular state or activity due to oxidative stress, often from exposure to high levels of reactive oxygen species.	0.10689	*EGFR* ↑ *GAS5* ↑	H1792
GO:0006979	GOBP_RESPONSE_TO_OXIDATIVE_STRESS	Processes that result in cellular activity changes as a response to elevated oxidative stress.	0.10689	*EGFR* ↑ *GAS5* ↑	H1792
GO:2000630	GOBP_POSITIVE_REGULATION_OF_MIRNA_METABOLIC_PROCESS	Involves cellular processes that activate or elevate frequency, rate, or extent of miRNA metabolic process.	0.10689	*EGFR* ↑ *GAS5* ↑	H1792
GO:0051254	GOBP_POSITIVE_REGULATION_OF_RNA_METABOLIC_PROCESS	Involves cellular processes that activate, or elevate the frequency of	0.10689	*EGFR* ↑ *GAS5* ↑	H1792
GO:0009894	GOBP_REGULATION_OF_CATABOLIC_PROCESS	Involves processes that modulate cellular catabolic processes	0.10689	*EGFR* ↑ *GAS5* ↑	H1792
GO:0098772	GOMF_MOLECULAR_FUNCTION_REGULATOR_ACTIVITY	Regulators in this term regulate activity of their targets via non-covalent binding, without inducing covalent modification to their targets.	0.10689	*EGFR* ↑ *GAS5* ↑	H1792
GO:0009057	GOBP_MACROMOLECULE_CATABOLIC_PROCESS	Chemical reactions resulting in the breakdown of high molecular mass particles.	0.10689	*EGFR* ↑ *GAS5* ↑	H1792
GO:0010586	GOBP_MIRNA_METABOLIC_PROCESS	Chemical pathways involving miRNAs, regulating gene expression.	0.10689	*EGFR* ↑ *GAS5* ↑	H1792
GO:0009719	GOBP_RESPONSE_TO_ENDOGENOUS_STIMULUS	Describes processes that result in cellular activity or state alteration due to a stimulus rising within the organism.	0.10689	*EGFR* ↑ *GAS5* ↑	H1792
GO:1901698	GOBP_RESPONSE_TO_NITROGEN_COMPOUND	Processes that result in cellular activity or state modification following the exposure of a nitrogen compound stimulus.	0.10689	*EGFR* ↑ *GAS5* ↑	H1792
GO:0034660	GOBP_NCRNA_METABOLIC_PROCESS	Includes chemical pathways and reactions in which non-coding RNA transcripts are involved.	0.10689	*EGFR* ↑ *GAS5* ↑	H1792

## Data Availability

The data presented in this study are openly available in the repository https://github.com/hasanalsharoh/vinonsclc.

## References

[1] Sung H., Ferlay J., Siegel R.L., Laversanne M., Soerjomataram I., Jemal A., Bray F. (2021). Global Cancer Statistics 2020: GLOBOCAN Estimates of Incidence and Mortality Worldwide for 36 Cancers in 185 Countries. CA Cancer J. Clin..

[2] Inamura K. (2017). Lung Cancer: Understanding Its Molecular Pathology and the 2015 WHO Classification. Front. Oncol..

[3] Jachowski A., Marcinkowski M., Szydłowski J., Grabarczyk O., Nogaj Z., Marcin Ł., Pławski A., Jagodziński P.P., Słowikowski B.K. (2023). Modern Therapies of Nonsmall Cell Lung Cancer. J. Appl. Genet..

[4] Zhou S., Yang H. (2023). Immunotherapy Resistance in Non-Small-Cell Lung Cancer: From Mechanism to Clinical Strategies. Front. Immunol..

[5] Xiao Y., Liu P., Wei J., Zhang X., Guo J., Lin Y. (2023). Recent Progress in Targeted Therapy for Non-Small Cell Lung Cancer. Front. Pharmacol..

[6] Zappa C., Mousa S.A. (2016). Non-Small Cell Lung Cancer: Current Treatment and Future Advances. Transl. Lung Cancer Res..

[7] Kang D.H., Kim J.O., Jung S.S., Park H.S., Chung C., Park D., Lee J.E. (2019). Efficacy of Vinorelbine Monotherapy as Third- or Further-Line Therapy in Patients with Advanced Non-Small-Cell Lung Cancer. Oncology.

[8] Nobili S., Lavacchi D., Perrone G., Vicini G., Tassi R., Landini I., Grosso A., Roviello G., Mazzanti R., Santomaggio C. (2020). Vinorelbine in Non-Small Cell Lung Cancer: Real-World Data From a Single-Institution Experience. Oncol. Res..

[9] Faller B.A., Pandit T.N. (2011). Safety and Efficacy of Vinorelbine in the Treatment of Non-Small Cell Lung Cancer. Clin. Med. Insights Oncol..

[10] Monnet I., de Cremoux H., Soulié P., Saltiel-Voisin S., Bekradda M., Saltiel J.-C., Brain E., Rixe O., Yataghene Y., Misset J.-L. (2002). Oxaliplatin plus Vinorelbine in Advanced Non-Small-Cell Lung Cancer: Final Results of a Multicenter Phase II Study. Ann. Oncol..

[11] Tian Z., Yao W. (2023). Chemotherapeutic Drugs for Soft Tissue Sarcomas: A Review. Front. Pharmacol..

[12] Capasso A. (2012). Vinorelbine in Cancer Therapy. Curr. Drug Targets.

[13] Jordan M.A., Thrower D., Wilson L. (1991). Mechanism of Inhibition of Cell Proliferation by Vinca Alkaloids. Cancer Res..

[14] Shi G., Cai L., Jiang W., Sui G. (2011). Effects of Navelbine and Docetaxel on Gene Expression in Human Lung Cancer Cell Lines. Cell Biochem. Biophys..

[15] Orlandi P., Di Desidero T., Salvia G., Muscatello B., Francia G., Bocci G. (2018). Metronomic Vinorelbine Is Directly Active on Non Small Cell Lung Cancer Cells and Sensitizes the EGFRL858R/T790M Cells to Reversible EGFR Tyrosine Kinase Inhibitors. Biochem. Pharmacol..

[16] Liu Z., Fu Q., Wang Y., Cui L., Zhang W., Teng Y., Yu P. (2021). Synergy between Vinorelbine and Afatinib in the Inhibition of Non-Small Cell Lung Cancer Progression by EGFR and P53 Signaling Pathways. Biomed. Pharmacother..

[17] Genova C., Rossi G., Pezzuto A., Valmadre G., Rijavec E., Biello F., Barletta G., Tagliamento M., Bello M.G.D., Boccardo S. (2019). P2.14-02 Interim Survival Analysis of Gefitinib Plus Vinorelbine in Advanced EGFR-Mutant Non-Small Cell Lung Cancer (Genoa Trial). J. Thorac. Oncol..

[18] Tada H., Mitsudomi T., Misumi T., Sugio K., Tsuboi M., Okamoto I., Iwamoto Y., Sakakura N., Sugawara S., Atagi S. (2022). Randomized Phase III Study of Gefitinib Versus Cisplatin Plus Vinorelbine for Patients With Resected Stage II-IIIA Non–Small-Cell Lung Cancer With EGFR Mutation (IMPACT). JCO.

[19] Ikeda S., Tsuboi M., Sakai K., Misumi T., Akamatsu H., Shoda H., Sakakura N., Nakamura A., Ohde Y., Hayashi H. (2023). NOTCH1 and CREBBP Co-Mutations Negatively Affect the Benefit of Adjuvant Therapy in Completely Resected EGFR-Mutated NSCLC: Translational Research of Phase III IMPACT Study. Mol. Oncol..

[20] Depenbrock H., Shirvani A., Rastetter J., Hanauske A.R. (1995). Effects of Vinorelbine on Epidermal Growth Factor-Receptor Binding of Human Breast Cancer Cell Lines in Vitro. Investig. New Drugs.

[21] Liu B., Zhao Y., Yang S. (2021). An Autophagy-Related Long Non-Coding RNA Prognostic Signature for Patients with Lung Squamous Carcinoma Based on Bioinformatics Analysis. Int. J. Gen. Med..

[22] Liu Y., Han Y., Zhang Y., Lv T., Peng X., Huang J. (2023). LncRNAs Has Been Identified as Regulators of Myeloid-Derived Suppressor Cells in Lung Cancer. Front. Immunol..

[23] Somasundaram K., Gupta B., Jain N., Jana S. (2022). LncRNAs Divide and Rule: The Master Regulators of Phase Separation. Front. Genet..

[24] Hussain M.S., Afzal O., Gupta G., Altamimi A.S.A., Almalki W.H., Alzarea S.I., Kazmi I., Fuloria N.K., Sekar M., Meenakshi D.U. (2023). Long Non-Coding RNAs in Lung Cancer: Unraveling the Molecular Modulators of MAPK Signaling. Pathol. Res. Pract..

[25] Maharati A., Moghbeli M. (2023). Long Non-Coding RNAs as the Critical Regulators of PI3K/AKT, TGF-β, and MAPK Signaling Pathways during Breast Tumor Progression. J. Transl. Med..

[26] Chakraborty J., Chakraborty S., Chakraborty S., Narayan M.N. (2023). Entanglement of MAPK Pathways with Gene Expression and Its Omnipresence in the Etiology for Cancer and Neurodegenerative Disorders. Biochim. Et Biophys. Acta Gene Regul. Mech..

[27] Paez J.G., Jänne P.A., Lee J.C., Tracy S., Greulich H., Gabriel S., Herman P., Kaye F.J., Lindeman N., Boggon T.J. (2004). EGFR Mutations in Lung Cancer: Correlation with Clinical Response to Gefitinib Therapy. Science.

[28] Tasharrofi B., Ghafouri-Fard S. (2018). Long Non-Coding RNAs as Regulators of the Mitogen-Activated Protein Kinase (MAPK) Pathway in Cancer. Klin. Onkol..

[29] Liu Z., Chen Q., Hann S.S. (2019). The Functions and Oncogenic Roles of CCAT1 in Human Cancer. Biomed. Pharmacother..

[30] Statello L., Guo C.-J., Chen L.-L., Huarte M. (2021). Gene Regulation by Long Non-Coding RNAs and Its Biological Functions. Nat. Rev. Mol. Cell Biol..

[31] Ji J., Dai X., Yeung S.-C.J., He X. (2019). The Role of Long Non-Coding RNA GAS5 in Cancers. Cancer Manag. Res..

[32] Berindan-Neagoe I., Chiorean R., Braicu C., Florian I.S., Leucuta D., Crisan D., Cocis A., Balacescu O., Irimie A. (2012). Quantitative mRNA Expression of Genes Involved in Angiogenesis, Coagulation and Inflammation in Multiforme Glioblastoma Tumoral Tissue versus Peritumoral Brain Tissue: Lack of Correlation with Clinical Data. Eur. Cytokine Netw..

[33] Welcome to Python.Org. https://www.python.org/.

[34] Anaconda|The World’s Most Popular Data Science Platform. https://www.anaconda.com/.

[35] Kluyver T., Ragan-Kelley B., Pérez F., Granger B.E., Bussonnier M., Frederic J., Kelley K., Hamrick J., Grout J., Corlay S. (2016). Jupyter Notebooks—A Publishing Format for Reproducible Computational Workflows. Positioning and Power in Academic Publishing: Players, Agents and Agendas.

[36] Fang Z., Liu X., Peltz G. (2023). GSEApy: A Comprehensive Package for Performing Gene Set Enrichment Analysis in Python. Bioinformatics.

[37] Chou C.-H., Shrestha S., Yang C.-D., Chang N.-W., Lin Y.-L., Liao K.-W., Huang W.-C., Sun T.-H., Tu S.-J., Lee W.-H. (2018). miRTarBase Update 2018: A Resource for Experimentally Validated microRNA-Target Interactions. Nucleic Acids Res..

[38] JASPAR: An Open-Access Database of Transcription Factor Binding Profiles. http://jaspar.genereg.net.

[39] Harris C.R., Millman K.J., van der Walt S.J., Gommers R., Virtanen P., Cournapeau D., Wieser E., Taylor J., Berg S., Smith N.J. (2020). Array Programming with NumPy. Nature.

[40] Virtanen P., Gommers R., Oliphant T.E., Haberland M., Reddy T., Cournapeau D., Burovski E., Peterson P., Weckesser W., Bright J. (2020). SciPy 1.0: Fundamental Algorithms for Scientific Computing in Python. Nat. Methods.

[41] Hunter J.D. (2007). Matplotlib: A 2D Graphics Environment. Comput. Sci. Eng..

[42] Waskom M.L. (2021). Seaborn: Statistical Data Visualization. J. Open Source Softw..

[43] Pandas—Python Data Analysis Library. https://pandas.pydata.org/about/citing.html.

[44] PubChem TFAP2A—Transcription Factor AP-2 Alpha (Human). https://pubchem.ncbi.nlm.nih.gov/gene/TFAP2A/human.

[45] Xiong Y., Feng Y., Zhao J., Lei J., Qiao T., Zhou Y., Lu Q., Jiang T., Jia L., Han Y. (2021). TFAP2A Potentiates Lung Adenocarcinoma Metastasis by a Novel miR-16 Family/TFAP2A/PSG9/TGF-β Signaling Pathway. Cell Death Dis..

[46] Zhu Q., Zhang Y., Li M., Zhang Y., Zhang H., Chen J., Liu Z., Yuan P., Yang Z., Wang X. (2023). MiR-124-3p Impedes the Metastasis of Non-Small Cell Lung Cancer via Extracellular Exosome Transport and Intracellular PI3K/AKT Signaling. Biomark. Res..

[47] Zhou X., Zhou R., Zhou H., Li Q., Hong J., Meng R., Zhu F., Zhang S., Dai X., Peng G. (2018). ETS-1 Induces Endothelial-Like Differentiation and Promotes Metastasis in Non-Small Cell Lung Cancer. Cell. Physiol. Biochem..

[48] Du J., Qian J., Zheng B., Xu G., Chen H., Chen C. (2021). miR-21-5p Is a Biomarker for Predicting Prognosis of Lung Adenocarcinoma by Regulating PIK3R1 Expression. Int. J. Gen. Med..

[49] Lorenz C., Hillmer A.M., Brägelmann J. (2023). Predicting the next Move: Tracking the Complexity of Lung Cancer Evolution and Metastasis. Signal Transduct. Target. Ther..

[50] Fan D.-P., Zhang Y.-M., Hu X.-C., Li J.-J., Zhang W. (2013). Activation of AKT/ERK Confers Non-Small Cell Lung Cancer Cells Resistance to Vinorelbine. Int. J. Clin. Exp. Pathol..

[51] Romitan M., Zanoaga O., Budisan L., Jurj A., Raduly L., Pop L., Ciocan C., Pirlog R., Braicu C., Ciuleanu T.E. (2023). MicroRNAs Expression Profile in Chemotherapy-Induced Cardiotoxicity in Non-Small Cell Lung Cancer Using a Co-Culture Model. Biomol. Biomed..

[52] Vardas V., Ju J.A., Christopoulou A., Xagara A., Georgoulias V., Kotsakis A., Alix-Panabières C., Martin S.S., Kallergi G. (2023). Functional Analysis of Viable Circulating Tumor Cells from Triple-Negative Breast Cancer Patients Using TetherChip Technology. Cells.

[53] Liu H., Fu Q., Lu Y., Zhang W., Yu P., Liu Z., Sun X. (2020). Anti-Tubulin Agent Vinorelbine Inhibits Metastasis of Cancer Cells by Regulating Epithelial-Mesenchymal Transition. Eur. J. Med. Chem..

[54] Dhyani P., Quispe C., Sharma E., Bahukhandi A., Sati P., Attri D.C., Szopa A., Sharifi-Rad J., Docea A.O., Mardare I. (2022). Anticancer Potential of Alkaloids: A Key Emphasis to Colchicine, Vinblastine, Vincristine, Vindesine, Vinorelbine and Vincamine. Cancer Cell Int..

[55] Xu B., Sun T., Wang S., Lin Y. (2021). Metronomic Therapy in Advanced Breast Cancer and NSCLC: Vinorelbine as a Paradigm of Recent Progress. Expert Rev. Anticancer Ther..

[56] Jin K., Chen B., Wang C., Zhang B., Zhang J., Kong M., Wang L., Zhu C., Shen J. (2021). Efficacy and Safety of Vinorelbine and Cisplatin Regimen of Different Doses and Intensities for Neoadjuvant Chemotherapy in Patients with Locally Advanced Esophageal Carcinoma. Ann. Transl. Med..

[57] Klotz D.M., Nelson S.A., Kroboth K., Newton I.P., Radulescu S., Ridgway R.A., Sansom O.J., Appleton P.L., Näthke I.S. (2012). The Microtubule Poison Vinorelbine Kills Cells Independently of Mitotic Arrest and Targets Cells Lacking the APC Tumour Suppressor More Effectively. J. Cell Sci..

[58] Simoens C., Lardon F., Pauwels B., De Pooter C.M., Lambrechts H.A., Pattyn G.G., Breillout F., Vermorken J.B. (2008). Comparative Study of the Radiosensitising and Cell Cycle Effects of Vinflunine and Vinorelbine, in Vitro. BMC Cancer.

[59] Ann Jordan M., Thrower D., Wilson L. (1992). Effects of Vinblastine, Podophyllotoxin and Nocodazole on Mitotic Spindles: Implications for the Role of Microtubule Dynamics in Mitosis. J. Cell Sci..

[60] Meraldi P., Draviam V.M., Sorger P.K. (2004). Timing and Checkpoints in the Regulation of Mitotic Progression. Dev. Cell.

[61] Bakhoum S.F., Kabeche L., Compton D.A., Powell S.N., Bastians H. (2017). Mitotic DNA Damage Response: At the Crossroads of Structural and Numerical Cancer Chromosome Instabilities. Trends Cancer.

[62] Busacca S., O’Regan L., Singh A., Sharkey A.J., Dawson A.G., Dzialo J., Parsons A., Kumar N., Schunselaar L.M., Guppy N. (2021). BRCA1/MAD2L1 Deficiency Disrupts the Spindle Assembly Checkpoint to Confer Vinorelbine Resistance in Mesothelioma. Mol. Cancer Ther..

[63] Di Martino M.T., Arbitrio M., Caracciolo D., Cordua A., Cuomo O., Grillone K., Riillo C., Caridà G., Scionti F., Labanca C. (2022). miR-221/222 as Biomarkers and Targets for Therapeutic Intervention on Cancer and Other Diseases: A Systematic Review. Mol. Ther. Nucleic Acids.

[64] Dong S., Qu X., Li W., Zhong X., Li P., Yang S., Chen X., Shao M., Zhang L. (2015). The Long Non-Coding RNA, GAS5, Enhances Gefitinib-Induced Cell Death in Innate EGFR Tyrosine Kinase Inhibitor-Resistant Lung Adenocarcinoma Cells with Wide-Type EGFR via Downregulation of the IGF-1R Expression. J. Hematol. Oncol..

[65] Ma J., Miao H., Zhang H., Ren J., Qu S., Da J., Xu F., Zhao H. (2021). LncRNA GAS5 Modulates the Progression of Non-Small Cell Lung Cancer through Repressing miR-221-3p and up-Regulating IRF2. Diagn. Pathol..

[66] Fu Y., Liu L., Zhan J., Zhan H., Qiu C. (2021). LncRNA GAS5 Expression in Non-Small Cell Lung Cancer Tissues and Its Correlation with Ki67 and EGFR. Am. J. Transl. Res..

[67] Zhou Y., Chen B. (2020). GAS5-mediated Regulation of Cell Signaling (Review). Mol. Med. Rep..

[68] Korrodi-Gregório L., Soto-Cerrato V., Vitorino R., Fardilha M., Pérez-Tomás R. (2016). From Proteomic Analysis to Potential Therapeutic Targets: Functional Profile of Two Lung Cancer Cell Lines, A549 and SW900, Widely Studied in Pre-Clinical Research. PLoS ONE.

[69] Martínez-Campa C., Casado P., Rodríguez R., Zuazua P., García-Pedrero J.M., Lazo P.S., Ramos S. (2006). Effect of Vinca Alkaloids on ERα Levels and Estradiol-Induced Responses in MCF-7 Cells. Breast Cancer Res. Treat..

[70] Xu H. (2022). 369P Comparison of the Efficacy and Safety of Icotinib with Vinorelbine or without Vinorelbine as First-Line Treatment for Advanced Lung Adenocarcinoma in Patients with Sensitive EGFR Mutations: A Retrospective Study. Ann. Oncol..

[71] Izumi H., Touge H., Igishi T., Makino H., Nishii-Ito S., Takata M., Nakazaki H., Ueda Y., Matsumoto S., Kodani M. (2015). Favorable Effect of the Combination of Vinorelbine and Dihydropyrimidine Dehydrogenase-inhibitory Fluoropyrimidine in EGFR-mutated Lung Adenocarcinoma: Retrospective and in Vitro Studies. Int. J. Oncol..

[72] Tsai C.-M., Chiu C.-H., Chang K.-T., Chen J.-T., Lai C.-L., Chen Y.-M., Hsiao S.-Y. (2012). Gefitinib Enhances Cytotoxicities of Antimicrotubule Agents in Non-Small-Cell Lung Cancer Cells Exhibiting No Sensitizing Epidermal Growth Factor Receptor Mutation. J. Thorac. Oncol..

[73] Dal Bello M.G., Alama A., Barletta G., Coco S., Truini A., Vanni I., Boccardo S., Genova C., Rijavec E., Biello F. (2015). Sequential Use of Vinorelbine Followed by Gefitinib Enhances the Antitumor Effect in NSCLC Cell Lines Poorly Responsive to Reversible EGFR Tyrosine Kinase Inhibitors. Int. J. Cancer.

[74] Fu Y., Liu L., Wu H., Zheng Y., Zhan H., Li L. (2023). LncRNA GAS5 Regulated by FTO-Mediated m6A Demethylation Promotes Autophagic Cell Death in NSCLC by Targeting UPF1/BRD4 Axis. Mol. Cell. Biochem..

[75] Zhou L., Jiang H., Lin L., Li Y., Li J. (2023). lncRNA GAS5 Suppression of the Malignant Phenotype of Ovarian Cancer via the miR-23a-WT1 Axis. Ann. Transl. Med..

[76] Alharbi K.S. (2023). Exploring GAS5′s Impact on Prostate Cancer: Recent Discoveries and Emerging Paradigms. Pathol. Res. Pract..

[77] Zhang T., Leng Y., Duan M., Li Z., Ma Y., Huang C., Shi Q., Wang Y., Wang C., Liu D. (2023). LncRNA GAS5-hnRNPK Axis Inhibited Ovarian Cancer Progression via Inhibition of AKT Signaling in Ovarian Cancer Cells. Discov. Oncol..

[78] Gao X., Lu C., Liu Z., Lin Y., Huang J., Lu L., Li S., Huang X., Tang M., Huang S. (2023). RBM38 Reverses Sorafenib Resistance in Hepatocellular Carcinoma Cells by Combining and Promoting lncRNA-GAS5. Cancers.

[79] Ye L., Shi H., Yu C., Fu J., Chen C., Wu S., Zhan T., Wang B., Zheng L. (2020). LncRNA Neat1 Positively Regulates MAPK Signaling and Is Involved in the Pathogenesis of Sjögren’s Syndrome. Int. Immunopharmacol..

[80] Campos-Parra A.D., López-Urrutia E., Orozco Moreno L.T., López-Camarillo C., Meza-Menchaca T., Figueroa González G., Bustamante Montes L.P., Pérez-Plasencia C. (2018). Long Non-Coding RNAs as New Master Regulators of Resistance to Systemic Treatments in Breast Cancer. Int. J. Mol. Sci..

[81] Chen L., Ren P., Zhang Y., Gong B., Yu D., Sun X. (2020). Long Non-Coding RNA GAS5 Increases the Radiosensitivity of A549 Cells through Interaction with the miR-21/PTEN/Akt Axis. Oncol. Rep..

[82] Zhang Y., Yang S.-H., Guo X.-L. (2017). New Insights into Vinca Alkaloids Resistance Mechanism and Circumvention in Lung Cancer. Biomed. Pharmacother..

